# Correlation and prognostic implications of intratumor and tumor draining lymph node Foxp3^+^ T regulatory cells in colorectal cancer

**DOI:** 10.1186/s12876-022-02205-0

**Published:** 2022-03-16

**Authors:** Bing Yan, Jianmei Xiong, Qianwen Ye, Tianhui Xue, Jia Xiang, Mingyue Xu, Fang Li, Wei Wen

**Affiliations:** 1 Department of Oncology, Hainan Hospital of Chinese PLA General Hospital, No. 80 of Jianglin Road, Haitang District of Sanya City, Hainan province 572000 People’s Republic of China; 2 Department of Neurology, Hainan Hospital of Chinese PLA General Hospital, No. 80 of Jianglin Road, Haitang District of Sanya City, Hainan Province 572000 People’s Republic of China; 3 Department of General Surgery, Hainan Hospital of Chinese PLA General Hospital, No. 80 of Jianglin Road, Haitang District of Sanya City, Hainan Province 572000 People’s Republic of China

**Keywords:** Colorectal cancer (CRC), T regulatory cells (Tregs), Tumor draining lymph nodes (TDLNs), Correlation, Survival

## Abstract

**Background:**

The prognostic value of intratumor T regulatory cells (Tregs) in colorectal cancer (CRC) was previously reported, but the role of these cells in tumor draining lymph nodes (TDLNs) was less addressed.

**Methods:**

A total of 150 CRC stages I-IV were retrospectively enrolled. Intratumor and TDLN Tregs were examined by immunohistochemical assay. The association of these cells was estimated by Pearson correlation. Survival analyses of subgroups were conducted by Kaplan–Meier curves, and the log-rank test and risk factors for survival were tested by the Cox proportional hazard model.

**Results:**

High accumulation of Tregs in tumors was significant in patients with younger age and good histological grade, where enrichment of these cells in TDLNs was more apparent in those with node-negative disease and early TNM stage disease, both of which were more common in early T stage cases. A significant correlation of intratumoral and TDLN Tregs was detected. Patients with higher intratumoral Tregs displayed significantly better PFS and OS than those with lower Tregs. However, no such differences were found, but a similar prognostic prediction trend was found for these cells in TDLNs. Finally, intratumoral Tregs were an independent prognostic factor for both PFS (HR = 0.97, 95% CI 0.95–0.99, *P* < 0.01) and OS (HR = 0.98, 95% CI 0.95–1.00, *P* = 0.04) in the patients.

**Conclusions:**

Higher intratumor Tregs were associated with better survival in CRC. Although no such role was found for these cells in TDLNs, the positive correlation and similar prognostic prediction trend with their intratumoral counterparts may indicate a parallelized function of these cells in CRC.

**Supplementary Information:**

The online version contains supplementary material available at 10.1186/s12876-022-02205-0.

## Introduction

Colorectal cancer (CRC) remains one of the main cancers worldwide, accounting for 9.8% of all new cases and 9.2% of all deaths for all cancers combined in 2021 [1]. In addition, the disease has become increasingly popular among young adults over the last 25 years [2]. Searching for a simple, reliable prognostic marker for the disease is thus of pivotal importance in practice.

T regulatory cells (Tregs), which are specifically labeled by forkhead box transcription Factor 3 (Foxp3) [3], are a small heterogeneous subset of CD4 + T cells. The main function of these cells was thought to block antitumor immune responses in cancer patients, and not surprisingly, increased counts of these cells in tumors could not only favor the development or growth of malignant cells but also influence the outcome of the patients [4]. The notorious role of these cells in predicting survival in malignancies was validated by a group of reports in gastric cancer [5], pancreatic cancer [6], breast cancer [7], ovarian cancer [8], and non-small cell lung cancer [9]. Nonetheless, the correlation of these cells with dismal outcome was not robustly established in CRC. Previously, although a series of reports concluded that the accumulation of intratumoral Tregs could predict good survival [10–13], there were also studies indicating that single Tregs were insufficient to predict prognosis [14–16]. To date, the paradox role of Tregs in predicting prognosis in CRC still needs further investigation.

Notably, Tregs were also found to be increased in other locations, such as peripheral blood in CRC [17]. TDLNs, which conventionally referred to pericolic, intermediate and additional main nodes for CRC patients underwent lymphadenectomy [18, 19], are an important sites where lymphocytes encounter tumor-specific antigens and generate antitumor immunity [20]. It was expected that the immune status of these nodes would have a profound effect on the spread of cancer [21]. Previously, the accumulation of Tregs was found to be significantly higher in TDLNs in many cancers [22–25] and was thought to promote cancer development. Similarly, in CRC, Gai et al. included 20 patients with metastatic TDLNs (mTDLNs) and 32 patients with metastasis-free TDLNs (mfTDLNs) and found that Tregs were significantly enriched in mTDLNs [26]; in line with this, Kazama et al. enrolled 50 patients and found that Tregs were enriched in regional lymph nodes, particularly in those near the lesions [27]. However, Deng et al. collected 10 patients with mTDLNs and 22 cases with mfTDLNs and found no such difference (only reported stage III cases), but they found that Tregs in TDLNs rather than those in tumors and peripheral blood were positively associated with disease stage [28]. However, these studies may be biased by their low number of cases and did not test the correlation of these cells with those in tumors or the individual prognostic value.

In this study, we aimed to detect Tregs in tumors as well as TDLNs and to determine the underlying correlation and their individual prognostic value. Furthermore, we compared their prognostic efficacy with other robust prognostic indicators, including the neutrophil to lymphocyte ratio (NLR), lymphocyte to monocyte ratio (LMR), and prognostic nutritional index (PNI), in CRC.

## Patients and methods

### Patient enrollments

From December 2012 to January 2018, CRC patients with radical recession of primary lesions in Hainan Hospital of Chinese PLA General Hospital were enrolled, and patients were excluded if they met any one of the following criteria: 1. neoadjuvant therapies; 2. missing laboratory tests within a week before surgery or key information in postoperative pathological reports; 3. multiple or recurrent malignancies or in situ lesions; or 4. loss to follow-up or less than 36 months. Other clinicopathological parameters, including body mass index (BMI) and tumor size (TS), were collected as previously reported [29–31]. The study was conducted in accordance with the principles stated in the Declaration of Helsinki and was approved by the ethics committee of Hainan Hospital of Chinese PLA General Hospital (ID: 301HLFYLS15), written informed consent was obtained from the patients or their authorized relatives.

### Immunohistochemical staining of Foxp3 in tumors and TDLNs

Resected fresh samples were immediately processed by standard histological methods, and the staining was carried out as follows. In brief, 5-µm slides were cut for both tumors (samples with necrosis and broken areas were excluded) and TDLNs, and one node was randomly selected either in cases with mTDLNs or mfTDLNs (including those with tumor deposits). After deparaffinization, sodium citrate solution was used for antigen retrieval for 20 min and then cooled to room temperature. Subsequently, 3% hydrogen peroxide was used for blocking for 10 min. After washing, nonspecific antigen blocking was then performed with 5% goat serum for 30 min at room temperature (cat. no. C0265; Beyotime Institute of Biotechnology). After washing, the primary rabbit anti-human Foxp3 antibody (dilution 1:50; cat. no. #98377; CST) was incubated at 4 °C overnight, and the negative control was conducted by replacement of the primary antibody with identically diluted 5% nonimmunized rabbit serum (cat. no. A7016, Beyotime Institute of Biotechnology). After washing, goat anti-rabbit IgG H&L (HRP)-preadsorbed secondary antibody (dilution 1:500, cat. no. ab7090; Abcam) were added for 30 min at room temperature. Staining was then achieved by adding 3,3′diaminobenzidine (DAB, cat. no. ab64238; Abcam) according to the manufacturer’s protocol, the slides were then washed and mounted. The results were read under a light microscope at a magnification of × 200 (BX51, Olympus Corporation) by two independent pathologists who were blinded to the clinical information. The absolute number of Tregs in the tumor and in TDLNs was counted as follows: for each slides, five hot spots in 10 high power field (HPF) with the highest number of positive cells were selected, and JPEG images were taken by using a digital camera, then the number of positive cells in the images were counted by using ImageJ software as previous report [32]; patients were then divided into low or high subgroups by the mean counts as previously reported [9, 11].

### Calculation of inflammation prognostic indicators

Routine laboratory data were collected as described in our previous report [31], and the NLR [33], LMR [34], and PNI [35] were estimated according to previous reports.

### Definition of progression-free survival (PFS) and overall survival (OS)

The follow-up was conducted as scheduled in a previous report [31]. PFS was defined as the date of operation to the point of first recurrence of any location, disease progression according to the RECIST (version 1.1) [36] or death from any cause. OS was defined as the point of operation to the date of any cause of death. The latest follow-up point was in June 2021.

### Statistical analysis

Statistical analyses were conducted by SPSS 20.0 (SPSS Inc., Chicago, IL, USA). Differences in clinicopathological parameters in Tregs-low or high in tumors and TDLNs were determined by χ2-test, Student’s t, or nonparametric rank sum test when appropriate. The association of Tregs in tumors and TDLNs, as well as with systemic inflammation markers, was determined by Pearson correlation. Survival differences for Treg-low or Treg-high groups were determined by Kaplan–Meier (K-M) analysis followed by log-rank tests. Risk factors for survival were estimated by a Cox proportional hazards model. Double-sided *P* < 0.05 was considered statistically significant.

## Results

### Patient characteristics and the differences in clinicopathological parameters in intratumoral and TDLN high or low Tregs

**Fig. 1:**
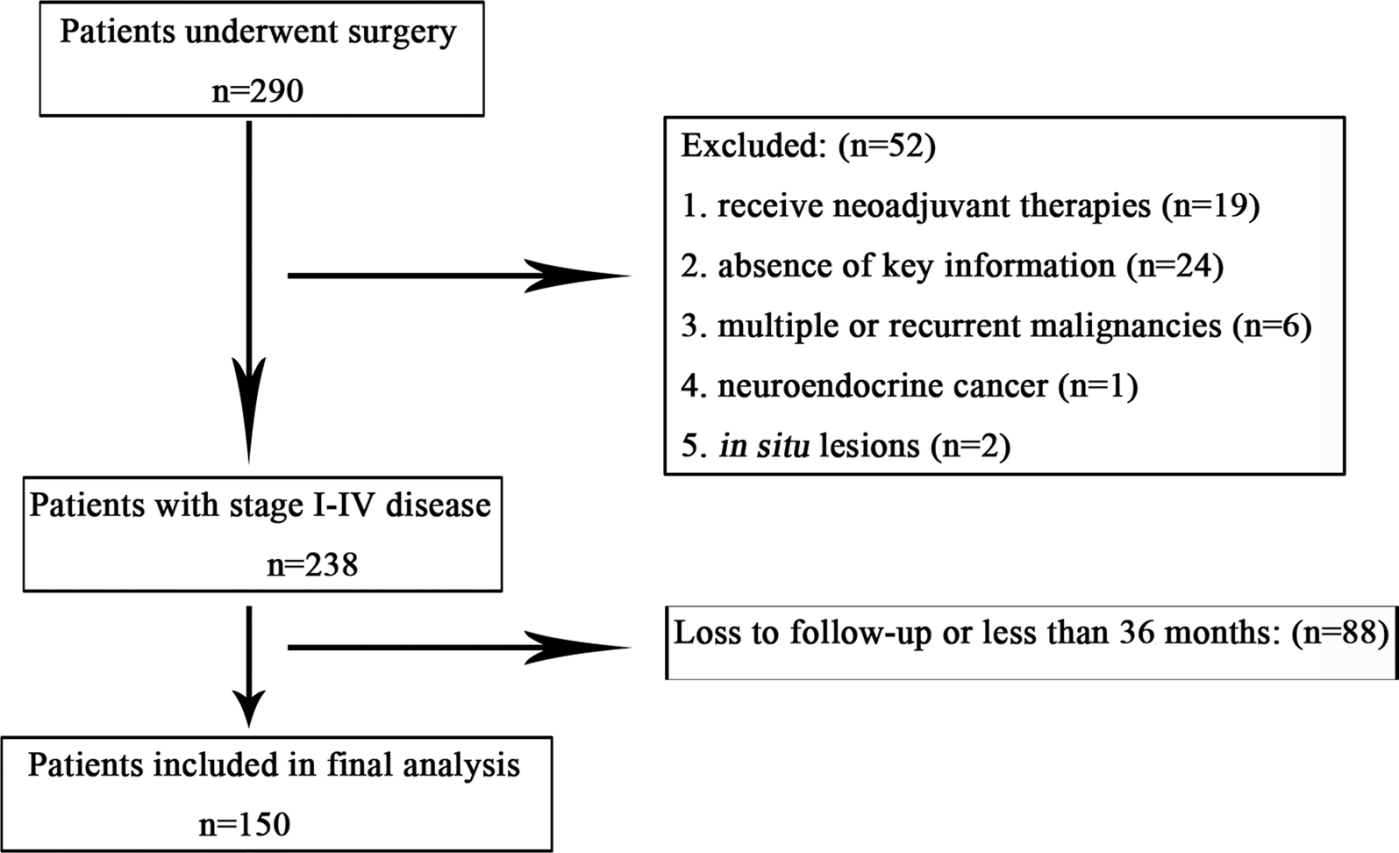
Flow diagram of the study

**Fig. 2:**
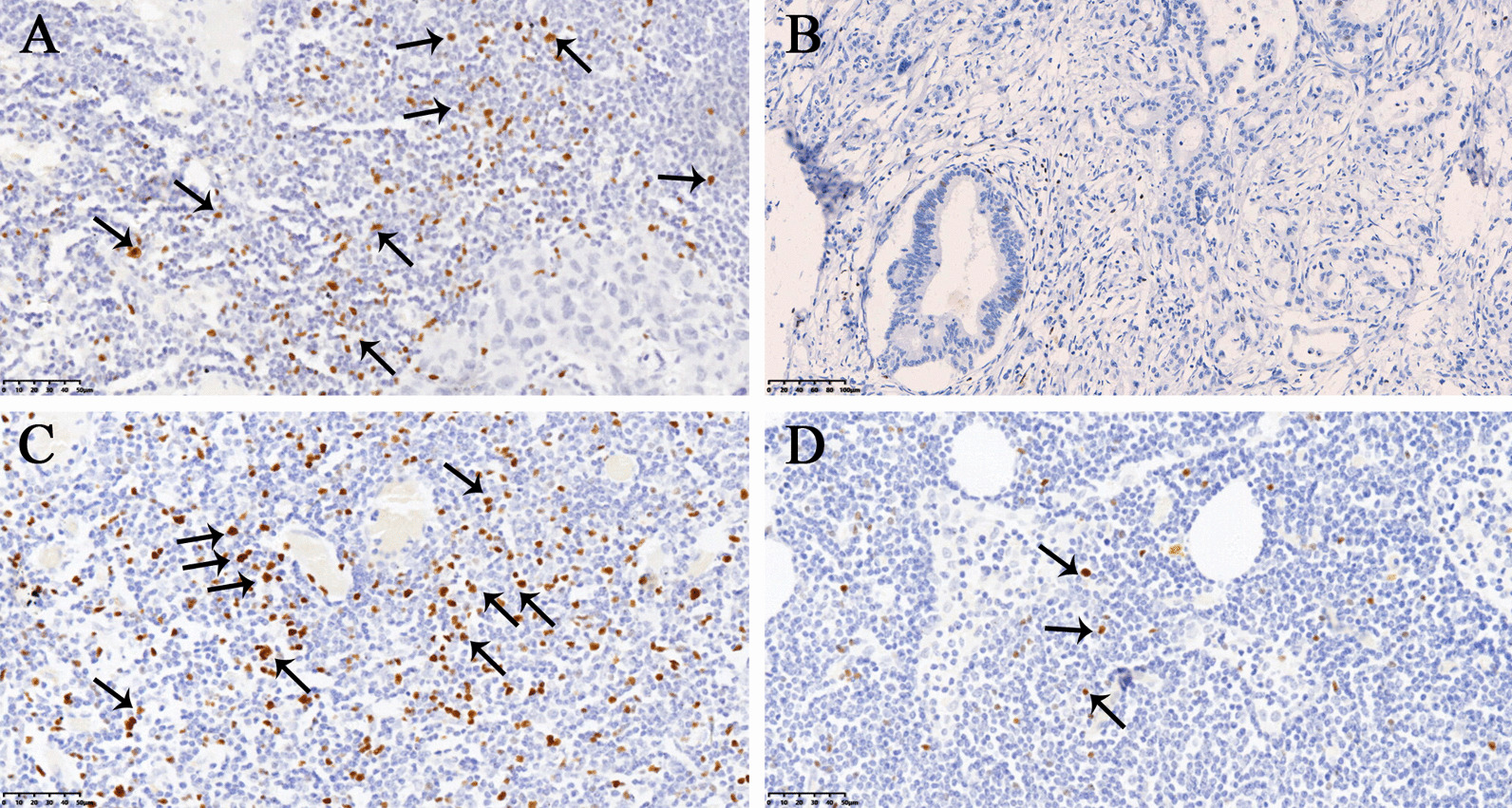
Accumulation of Tregs in tumor and in mTDLNs. **A** High Tregs in tumor; **B** No obvious Tregs in tumor; **C** High Tregs in mTDLNs; **D** Low Tregs in mTDLNs. Positive Tregs are indicated by black arrows with a magnification × 200

In total, 290 patients were enrolled, and 150 patients (94 males, 56 females) were included in the final analysis (Fig. 1). The mean age of the patients was 60.36 y (range: 24–85 y), and the mean follow-up was 47.61 m (range 1–102 m). A total of 2183 nodes were harvested with 279 mTDLNs. In addition, three patients with mfTDLNs and 10 patients with mTDLNs presented tumor deposits. Tregs were differently expressed in intratumoral and mTDLNs (Fig. 2), the number of the Tregs ranged from 0 to 110.0 (mean: 20.03), 0 to 260 (mean: 43.49) per HPF respectively. And approximately 1/3 (50/150 and 49/150, respectively) of patients displayed intratumoral Tregs and TDLN-high Tregs. As shown in Table 1, Tregs with high intratumoral frequencies were significantly more obvious in younger and good histological grade patients (both *P* < 0.01), where TDLNs with high frequencies were more apparent in node-negative (*P* < 0.01) and early TNM stage (*P* = 0.02) patients, and both of which were more common in early T stages (*P* < 0.01 and *P* = 0.03, respectively).

**Table 1:** Comparison of differences for the clinicopathological parameters in low or high Foxp3^+^ Tregs intratumor or TDLNs

	Intratumor			TDLNs		
	Low	High	*P*	Low	High	*P*
Age (years)			**< 0.01**			0.17
< 60	36	31		41	26	
≥ 60	64	19		60	23	
Gender			1.00			0.47
Female	37	19		40	16	
Male	63	31		61	33	
Tumor location			0.41			0.15
Right side	21	14		20	15	
Left side	79	36		81	34	
Histological grade			**< 0.01**			0.65
Well + moderate	77	48		83	42	
Poor	23	2		18	7	
CEA status			0.16			0.59
Normal	57	35		60	32	
Elevated	43	15		41	17	
Invasive depth			**< 0.01**			**0.03**
T_1+2_	14	21		18	17	
T_3+4_	86	29		83	32	
Tumor diameter (cm)			0.22			0.48
< 4	37	24		39	22	
≥ 4	63	26		62	27	
Node involvement(s)			0.73			**< 0.01**
N0	56	30		50	36	
N_1+2_	44	20		51	13	
Distant metastasis			1.00			0.27
M_0_	94	47		93	48	
M_1_	6	3		8	1	
TNM stages			0.49			**0.02**
I + II	53	30		49	34	
III + IV	47	20		52	15	
BMI (kg/m^2^)	23.48 ± 3.86	23.65 ± 3.69	0.78	23.53 ± 3.45	23.56 ± 4.47	0.96
Pre-operative measurements						
NLR	2.53 ± 2.80	2.32 ± 1.23	0.63	2.34 ± 1.27	2.71 ± 3.78	0.13
LMR	3.89 ± 1.53	4.34 ± 1.79	0.11	3.95 ± 1.61	4.24 ± 1.67	0.30
PNI	48.60 ± 6.12	48.33 ± 5.63	0.79	48.34 ± 6.07	48.87 ± 5.70	0.60

Statistical differences were shown in bold

### Correlation of intratumor and TDLN Tregs and prognostic inflammation indicators

By Pearson correlation analysis, a significant correlation was found for intratumor and TDLN Tregs. In addition, intratumoral Tregs were also positively correlated with preoperative LMR and TS; however, no such correlation was found for TDLN Tregs (Table 2).

**Table 2:** Correlation of intratumor and TDLNs Tregs with NLR, LMR, PNI and TS

	Intratumor	TDLNs	NLR	LMR	PNI	TS
Intratumor		R = 0.16	R = − 0.09	R = 0.29	R = 0.04	R = − 0.21
		***P* = 0.04**	*P* = 0.29	***P* < 0.01**	*P* = 0.63	***P* = 0.01**
TDLNs	R = 0.16		R = − 0.03	R = 0.07	R = 0.01	R = − 0.01
	***P* = 0.04**		*P* = 0.72	*P* = 0.40	*P* = 0.97	*P* = 0.87

Statistical differences were shown in bold

### Survival differences of intratumor or TDLN high- or low-Treg subgroups in PFS and OS

**Fig. 3:**
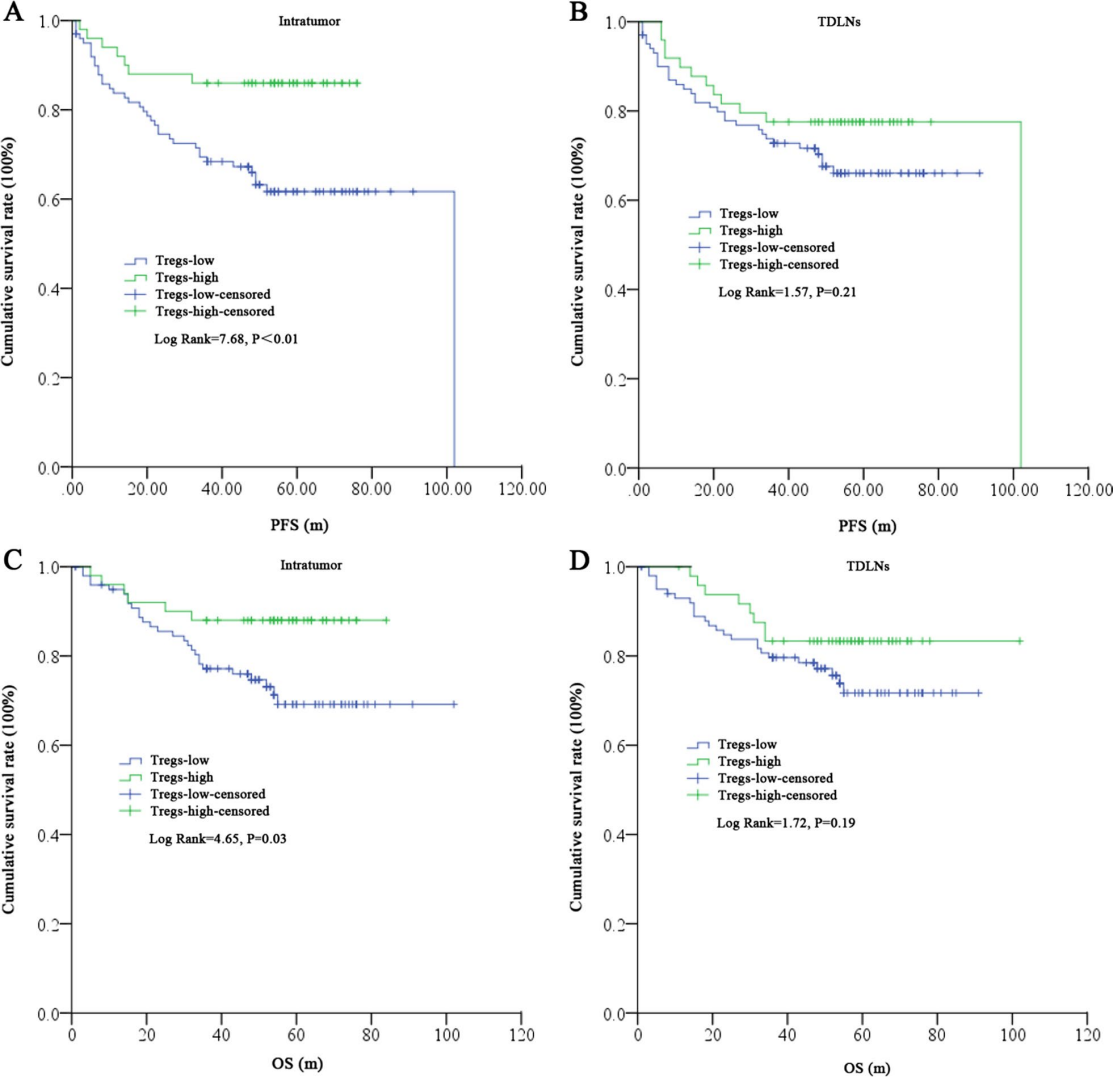
Prognostic role of Tregs in tumors and in TDLNs for PFS and OS. A, C. High Tregs in tumors predict superior PFS (**A**) and OS (**C**); **B**, **D** High or low Tregs in TDLNs display no significant differences in PFS (**B**) and OS (**D**)

Significant differences in intratumoral high and low Tregs in the 3-year PFS (14.00% vs*.* 37.00%, *P* < 0.01) and OS (12.00% vs. 27.00%, *P* = 0.04) rates could be detected, but no such differences could be found for TDLN Tregs in PFS (24.49% vs*.* 31.68%, *P* = 0.45) and OS (16.33% vs*.* 24.75%, *P* = 0.30). By K-M analyses, patients with intratumor high Tregs had a significantly better PFS (Tregs-high vs*.* low: 51.56 ± 18.75 m vs*.* 43.00 ± 24.61 m, Log rank = 7.68, *P* < 0.01) and OS (Tregs-high vs*.* low: 53.58 ± 17.30 m vs*.* 46.95 ± 21.94 m, Log rank = 4.65, *P* = 0.03) than those with Tregs-low (Fig. 3A, C). In addition, although patients with Tregs with high or low TDLNs did not show such differences in PFS (Tregs-high vs*.* low: 49.88 ± 21.72 m vs*.* 43.90 ± 23.62 m, Log rank = 1.57, *P* = 0.21) and OS (Tregs-high vs*.* low: 53.45 ± 18.10 m vs*.* 47.08 ± 21.62 m, Log rank = 1.72, *P* = 0.19), a similar survival difference trend could be found (Fig. 3B, D).

### Univariate and multivariate analyses of the prognostic risks for PFS and OS

Univariate tests indicated that CEA status, invasive depth, node involvement, distant metastasis, TNM stages, preoperative LMR and PNI, and intratumor Tregs were significant prognostic factors for PFS (Table 3) and OS (excluding preoperative LMR) (Table 4), and when all these factors (only those *P* < 0.05 in Tables 3, 4) were included in multivariate tests, the results indicated that intratumor Tregs were an independent prognostic factor for both PFS (HR = 0.97, 95% CI 0.95–0.99, *P* < 0.01) and OS (HR = 0.98, 95% CI 0.95–1.00, *P* = 0.04).

**Table 3:** Univariate and multivariate analyses of different parameters for PFS

	Univariate			Multivariate		
	*P*	HR	95% CI	*P*	HR	95% CI
Age (years)						
< 60	1					
≥ 60	0.13	1.63	0.87–3.06			
Gender						
Female	1					
Male	0.48	1.26	0.67–2.39			
Tumor location						
Right	1					
Left	0.08	0.46	0.20–1.10			
Histological grade						
Well + moderate	1					
Poor	0.29	1.49	0.71–3.11			
CEA status						
Normal	1					
Elevated	**0.02**	2.11	1.16–3.85			
Invasive depth						
T_1+2_	1					
T_3+4_	**0.02**	3.52	1.26–9.86			
Tumor diameter (cm)						
< 4	1					
≥ 4	0.36	1.34	0.72–2.51			
Node involvement						
N_0_	1					
N_1+2_	**< 0.01**	2.27	1.24–4.16			
Distant metastasis						
M0	1			1		
M1	**< 0.01**	10.75	4.87–23.73	**< 0.01**	8.71	3.88–19.49
TNM stage						
I + II	1					
II + IV	**< 0.01**	2.62	1.41–4.88			
BMI (kg/m^2^)	0.23	0.95	0.86–1.04			
Preoperative measures						
NLR	0.09	1.07	0.99–1.15			
LMR	**0.04**	0.81	0.66–0.99			
PNI	**< 0.01**	0.92	0.88–0.97	**< 0.01**	0.94	0.89–0.98
Tregs intratumor	**< 0.01**	0.97	0.95–0.99	**< 0.01**	0.97	0.95–0.99
Tregs in TDLNs	0.77	1.00	0.99–1.01			

Statistical differences were shown in bold

**Table 4:** Univariate and multivariate analyses of different parameters for OS

	Univariate			Multivariate		
	*P*	HR	95%CI	*P*	HR	95%CI
Age (years)						
< 60	1					
≥ 60	0.05	2.07	0.89–4.36			
Gender						
Female	1					
Male	0.39	1.38	0.66–2.90			
Tumor location						
Right	1					
Left	0.19	0.53	0.21–1.38			
Histological grade						
Well + moderate	1					
Poor	0.15	1.80	0.81–3.98			
CEA status						
Normal	1			1		
Elevated	**< 0.01**	2.59	1.30–5.18	**0.05**	2.04	1.00–4.15
Invasive depth						
T_1+2_	1					
T_3+4_	**0.02**	5.62	1.34–23.50			
Tumor diameter (cm)						
< 4	1					
≥ 4	0.09	1.93	0.90–4.15			
Node involvement						
N_0_	1			1		
N_1+2_	**0.01**	2.47	1.23–4.96	**0.02**	2.31	1.14–4.71
Distant metastasis						
M0	1			1		
M1	**< 0.01**	10.84	4.77–24.61	**< 0.01**	6.85	2.95–15.91
TNM stage						
I + II	1					
II + IV	**< 0.01**	3.01	1.46–6.21			
BMI (kg/m^2^)	0.07	0.91	0.82–1.01			
Preoperative measures						
NLR	0.11	1.07	0.99–1.16			
LMR	0.14	0.84	0.66–1.06			
PNI	**< 0.01**	0.92	0.87–0.96	**< 0.01**	0.92	0.87–0.98
Tregs intratumor	**0.03**	0.98	0.95–1.00	**0.04**	0.98	0.95–1.00
Tregs in TDLNs	0.77	1.00	0.99–1.01			

Statistical differences were shown in bold

## Discussion

In the present study, we found that Tregs accumulated differently in tumors and TDLNs. Although only intratumoral Tregs showed a significant prognostic value for patient survival, a positive correlation of these cells was established for those located in tumors and TDLNs; in addition, a similar prognostic trend of these cells in TDLNs as in tumors was observed. To the best of our knowledge, our study includes the largest sample to explore Tregs in TDLNs and examine their prognostic role as well as correlation with their intratumoral counterparts in CRC.

Taking into consideration its negative role in manipulating antitumor immune responses, the accumulation of Tregs is regarded as an indicator of poor survival in many cancers [5–9]. Commonly, enrichment of these cells is also associated with clinicopathological parameters such as node metastasis and vascular, lymphatic, or perineural invasion [37–39]. However, the notorious role of Tregs in survival prediction is still a matter of debate in CRC. For example, Katz et al. included 188 patients who underwent resection of liver metastases and found that single Tregs were not sufficient to predict recurrence-free survival [14]. Sinicrope et al. collected 160 stage II-III patients and found that Tregs were not prognostic [16]. However, studies with large samples yield different conclusions. For example, Frey et al. included 1420 patients staged I-III and found that high frequency intratumoral Tregs were associated with early T stage and improved 5-year survival rate and Tregs were independent prognostic factor mismatch-repair (MMR)-proficient cases [40]. Salama et al. enrolled 967 staged II-III patients and found that a high density of Tregs correlated with improved survival [41]. Our study also supports that the accumulation of Tregs in tumors suggests superior survival in CRC, which is in line with previous reports [40, 41]. To date, although studies robustly support the positive role of Tregs in prognosis in CRC, the underlying mechanism is still poorly understood. To this end, Ladoire et al. argued that Tregs could attenuate Th17 cell-dependent proinflammatory and tumor-enhancing responses, the latter of which was important in manipulating cancer cell growth, constituting a possible explanation for their favorable role in CRC prognosis [42]. In addition, Saito et al. showed that Tregs in tumors can be classified into Foxp3^hi^ and Foxp3^lo^ subsets, and the latter could release inflammatory cytokines and correlate with better prognosis in CRC [43]. In addition, Lin et al. found that there are different subsets of Tregs in CRC, named activated Tregs (Foxp3^hi^CD45RA^−^), nonsuppressive Tregs (Foxp3^lo^CD45RA^−^), and resting Tregs (Foxp3^lo^CD45RA^+^); only activated Tregs correlated with tumor metastases [44]. Nonetheless, these studies are not conclusive, and more studies are needed in the future.

TDLNs play an important role in inhibiting the spread of cancer [20, 21] and have been found to be of pivotal importance in immunotherapy in recent years [45]. Tregs were found to be clustered in TDLNs in previous studies in cancer patients [46, 47]. Theoretically, these cells should be positively correlated with those in tumors in terms of quantity and function [48, 49], such as neutrophils [50]. In fact, some reports in gastric cancer supported this notion; for example, Maruyama et al. found that Tregs were significantly increased in mTDLNs [22], and Lee et al. reported that high Treg density in sentinel lymph nodes (SLNs) was significantly associated with the metastasis of non-SLNs [21]. Interestingly, they also reported that the accumulation of Tregs in N1 was significantly higher than that in N2 and Nc (nonregional control nodes) [21]. Additionally, Kawaida et al. found that Tregs in N1 cases were significantly higher than those in control mesenteric lymph nodes or N2 cases [23]. All these results indicated that Tregs in TDLNs may promote cancer development, which is in line with intratumoral Tregs in prognosis to some extent. Of note, the aforementioned two studies in CRC [26, 28] concerning Tregs in TDLNs indicated a similar role of these cells in gastric cancer [21, 22], which is discordant with reports that support Tregs as a good indicator for survival in tumors in CRC [10–13]. We speculate that these reports may be biased by the limited sample size [26, 28], and in fact, there was also a report demonstrating that low Tregs in tumor-free SLNs are associated with node metastases in CRC [51]. In our study, we found a positive correlation of Tregs in TDLNs and in tumors, which is in line with the report in neutrophils (R = 0.28, *P* < 0.01) [50]. Additionally, consistent with Lee et al.’s report [19], we also found that Tregs were significantly enriched in N1 compared to N0 and N2 (*P* = 0.01 and *P* = 0.07, respectively, data not shown). More importantly, we detected that these cells presented a parallel prognostic trend to the intratumoral ones, which supports our aforementioned speculation that these cells may functionally correlate in CRC irrespective of their different localization.

It was reported that Tregs could not only release inflammatory cytokines such as interleukin (IL)-10, IL-35, tumor necrosis factor-α (TNF-α), and interferon-γ (INF-γ) [44, 52] but also suppress other cytokine-producing cells such as myeloid dendritic cells [53]. Some of these cytokines would have a profound effect on the control of the development of CRC. For example, it was found that high concentration of IL-35 in CRC could inhibit the cancer cell migration, invasion and proliferation, more importantly, suppress the cancer stem cells [54]; also, the concentration of TNF-α was found to be negatively correlated with the stage in CRC [55]. In addition, it was long-time established that TNF-α and INF-γ have a strong effect in inhibiting colon cancer cell proliferation [56], and a combination of these cytokines could resulted in 30–40% more growth inhibition in CRC cell lines [57]. All these evidence would to some extent support that a high accumulation of Tregs in tumor would have a positive role in prognosis. Except these, the NLR, LMR, and PNI were newly established prognostic indicators in recent years and could be manipulated by inflammatory cytokines [58, 59] released by cancer cells or other cells, such as Tregs. In our study, we found a significant correlation of intratumoral Tregs with LMR, and the latter was also positively associated with prognosis in CRC [60]. Tregs themselves are an important constituent of lymphocytes (which are significantly increased in CRC [17]), and their secretion of IL-10 could manipulate monocytes [59], which could then influence the LMR. However, the exact mechanisms of the relationship of these cells with LMR but no other markers are still poorly understood when taking into consideration the complex cellular/molecular network orchestrated by these cells in tumors or in TDLNs [52].

Notably, we found that 17.33% (26/150), 12% (18/150) cases were blank for Tregs in tumor and TDLNs, respectively. In fact, absence of Tregs in tumor has been registered in previous reports [61–63]. As a previous study indicated that hypoxic tumor microenvironment (TME) could result in a significant metabolic reprogramming for Tregs (which are especially susceptible to hypoxic metabolic signaling) and subsequently lead to an abnormal survival and proliferation of these cells [64]. Except this, it was found that certain inflammatory cytokines like IL-6, which was significantly higher in TME and peripheral blood of CRC patients [65, 66], could impact the migration capacity of Tregs to the lesion [67]. Take into account these studies, we speculate that the cases with blank Tregs would have a special TME or significant high IL-6 that could prevent the infiltrating or kill Tregs; however, more studies are still needed to validate this speculation.

Our study has some limitations: first, its retrospective nature cannot completely exclude confounding factors; second, patients with T3 or higher disease with other risk factors or those with mTDLNs would receive subsequent therapies after surgery, which would affect not only survival but also Tregs [68]; third, the information of some important genetic alternations was not available, in particular the RAS mutations, as a report indicated that such changes could affect TNF-induced apoptosis in CRC [69], which could intervention of the role of Tregs in CRC; fourth, although there were no differences in survival for Tregs in MMR-proficient or MMR-deficient patients [40, 70], these results were not validated in patients with mTDLNs or mfTDLNs, and large-sample studies with definite MMR status could resolve these questions in the future.

## Conclusion

Overall, our study indicated that higher intratumoral Tregs were associated with better survival in CRC, and a positive correlation of these cells in TDLNs could be found with intratumor Tregs. The similar prognostic prediction trend for these cells in TDLNs as intratumor Tregs suggested that these cells may share a parallelized function in patients; however, more studies are still needed in the future.

## Supplementary Information

**Additional file 1.** Dataset to generate the results of the study.

## Data Availability

All data generated or analyzed during this study are included in this published article and its Additional file 1.

## Abbreviations

CRC: Colorectal cancer
Tregs: T regulatory cells
Foxp3: Forkhead box transcription Factor 3
TDLNs: Tumor draining lymph nodes
PFS: Progression free survival
OS: Overall survival
NLR: Neutrophil to lymphocyte ratio
LMR: Lymphocyte to monocyte ratio
PNI: Prognostic nutritional index
BMI: Body mass index
TS: Tumor size
HPF: High power field
K-M: Kaplan–Meier analysis
CEA: Carcinoembryonic antigen
MMR: Mismatch-repair
